# Damage Caused by Material Defects of Carbon Composites Used on Various Types of Railway Pantographs

**DOI:** 10.3390/ma16051839

**Published:** 2023-02-23

**Authors:** Małgorzata Kuźnar

**Affiliations:** Department of Rail Vehicles and Transport, Cracow University of Technology, 31-878 Cracow, Poland; malgorzata.kuznar@pk.edu.pl; Tel.: +48-123-743-659

**Keywords:** pantograph sliding strip, predictive maintenance, sliding strip material, carbon strips, graphite composite, material damage, material defects, rail vehicle, current collector, pantograph plate, contact plate

## Abstract

Mainstream materials of the railway pantograph strips are carbon composites. They are subject to wear during use, as well as various types of damage. It is important that their operation time is as long as possible and that they are not damaged, as it may damage the remaining elements of the pantograph and the overhead contact line. As part of the article, three types of pantographs were tested: AKP−4E, 5ZL, and 150 DSA. They had carbon sliding strips made of MY7A2 material. By testing the same material on different types of current collectors, it was possible to check what impact the wear and damage of the sliding strips has on (among others) the method of their installation, i.e., whether the damage to the strips depends on the type of current collector and what is the participation of damage caused by material defects. As a result of the research, it was found that the type of pantograph on which it is used has an undoubted influence on the damage characteristics of the carbon sliding strips, whereas the damage caused by material defects can be classified as a more general group—the group of damage of a sliding strip, which also includes overburning of a carbon sliding strip.

## 1. Introduction

The sliding strip is the most frequently replaced element of the pantograph. The reasons for such a replacement are described in this article, with particular emphasis on damage caused by material defects. As part of the work, tests of sliding strips mounted on three types of pantographs were carried out. The collected data were preprocessed, decision rules were developed to enable further data analysis, and then representative data sets were selected. An analysis of variance between datasets concerning the reason for the replacement of the sliding strips was performed, box and whisker charts were used to show the thickness of the overlay during its replacement with a new one; depending on the reasons for replacement, the relationship between the thickness of the overlay and the reason for replacement was analyzed using Pareto–Lorenz diagrams and descriptive statistics of the discussed cases were made. The tests were aimed, among others, to determine whether the type of pantograph affects the damage characteristics of the sliding pads in relation to material defects.

To bring the subject of pantograph sliding strips closer, this section describes the construction of a current collector and contact strips, as well as the process of wear of carbon sliding strips.

### 1.1. Construction of a Current Collector, Contact Strips, Diagrams

In the European Union, the issues of the railway power supply system and its connections with others are described in Directive 2016/797 [1] on the “Interoperability of the rail system within the European Union”—TSI (Technical Specification for Interoperability). Related to them are subject standards (EN) and other national requirements for interoperability. Whereas the so-called interoperability constituents (elements on which the interoperability of the trans-European system depends directly or indirectly) are overhead contact lines, pantographs, and contact strips. The aforementioned directive also deals with the methodology of research and evaluation of the quality of the interaction between the current collector and the overhead contact line.

The European standard PL−EN 50206−1: 2010 [2] defines a pantograph as a device that receives current from one or two contact wires. The current collector consists of a base frame, a drive unit, an articulated frame, and a slider with contact strips. The pantograph changes its geometry. It is fully or partially energized in the working position. The pantograph is electrically insulated from the locomotive roof by an interface (insulator). It enables the transmission of electricity from the catenary to the vehicle’s electrical system.

Current collectors cooperating with the overhead contact line suspended above the track. Depending on the power supply system and the required operating conditions, they can be divided into:

- Symmetrical: diamond-shaped, cross-arm.
- Asymmetrical: single-arm.

**Figure 1:**
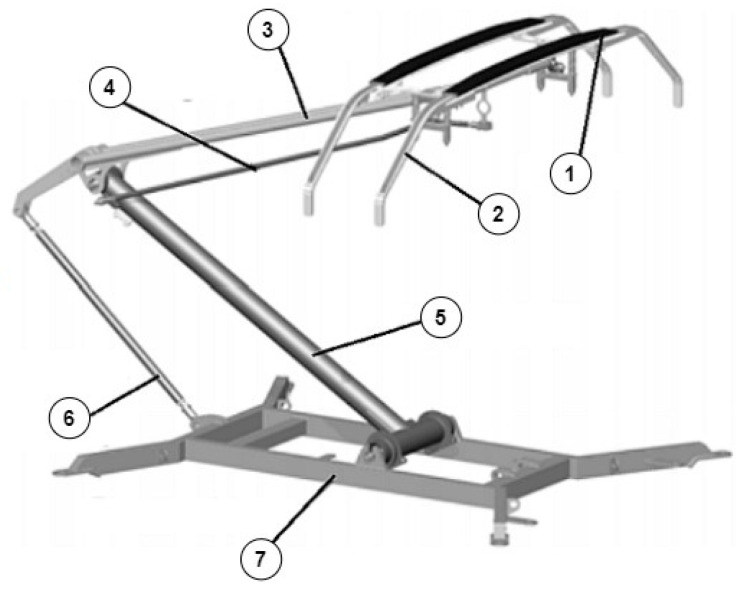
Scheme of single-arm pantograph where: 1—contact strip (sliding strip); 2—collector head; 3—upper arm and collector head guide; 4—upper guide rod; 5—lower arm; 6—lower guide rod; 7—base frame.

The construction of the pantograph (Figure 1) consists of: a base frame and a light articulated frame connected to it, on which in the upper arm a pivoting and spring−loaded slider or a set of sliding strips is placed. It is this element that works with the contact wires and creates one contact with the wire. The difference in individual solutions of the collectors concerns the method of suspending or springing the slider in relation to the upper arm of the frame, the shape of the frames, the method of designing the drive system, the material of the contact strips, additional damping, and securing devices.

The drive system of the current collector is a device that enables its operation. It generates the required constant or abruptly variable force of static pressure of the slider on the contact wires by the force of the system of stretched springs or by the force of the pneumatic (bellows) actuator.

The slider suspension is an element of the collector that reduces the influence of the slider mass on the variable component of the contact force. The slider is connected to the articulated frame via a system of spiral or flat springs. Depending on the value of the rated current of the collector, single or double slider systems with 2, 3, and 4 contact strips are used. The construction of the slider can be compact or in the form of two slats connected by a beam.

When comparing the half and symmetrical collectors, it can be seen that the half collectors have fewer moving members and hence less articulation and less frictional force. However, the aerodynamic force of these collectors depends on the direction of travel. In some designs, at high speeds, it is insufficient, and then special measures are applied to increase the aerodynamic force by adding an additional element in the upper joint or on the articulated frame or periodically increasing the contact force. Their disadvantage is that they are less stiff in the transverse direction. The challenges that the pantograph-catenary systems face are described in [3,4,5,6]. The pantograph catenary is under harsh working environments, such as the vehicle-track perturbations and such cases are modeled in [3], the high-speed flow is examined in [4], the impact of overhead line irregularity on current collection is described in [5], and the wind deflection analysis is tested in [6].

Due to the requirements of Directive 2016/797 and the TSI specification and the relevant standards, it is required that the current collector transfers current from the overhead contact line to the circuits of the rail vehicle:

− without detachment from the contact wires;
− lifting them above a predetermined value;
− wear as little as possible on the contact wire and its own contact strips.

At the same time, the current collector should ensure the current transmission of the desired intensity without excessive heating of the contact wires and the collector elements in the entire speed range and when stationary, regardless of the direction of travel. It should be remotely controlled and intended for the highest driving speeds, it must have a device that automatically drops the current collector during a failure. The static force generated by the drive system must be within the assumed range.

One of the most frequently used current collectors in Poland is the AKP4E and 5ZL collectors, successively replaced by the DSA150 collector. Technical parameters of the mentioned collectors are presented in Table 1 which was made on the basis of the information contained in the article [7] and the manufacturer’s materials [8].

### 1.2. The Process of Wear of Carbon Sliding Strips

During normal use of the current collector, the contact strip wears out naturally. The graphite contact strip wear forms primarily include mechanical wear, electrical wear, and material erosion, among which mechanical wear is the main form [9]. In this paper, only mechanical wear is considered. If the thickness of the sliding strip is smaller than the value assumed in the measurement card, the sliding strip should be replaced with a new one. Since the graphite contact strip interacts with the overhead contact line through sliding friction contact, the mechanical wear of the pantograph is directly related to the contact force, the contact wire, and the material and spatial relationships of the graphite contact strip. Adhesive wear is the most common type of mechanical wear in the pantograph-catenary (P−C) system [10]. No matter how well the surface is finished, its micro appearance is still roughened and covered with randomly placed bumps. When two bodies are in frictional contact, the higher roughnesses are compressed first, leading to stress concentrations due to the small contact area. When the contact force exceeds the yield strength of a body made of a softer material, relative sliding movement or even cracking between the unevenness and the surface causes wear to occur [11].

The operation lifetime of the sintered alloy strip can reach a value corresponding to an accumulated travel distance of or larger than 100,000 km, whereas those of the carbon and metal-impregnated carbon sliders are approximately 70,000–80,000 km and only approximately 60,000–70,000 km (or smaller), respectively [12]. Many companies prefer to replace the strips a bit earlier to avoid a situation where exceeding the limit value can cause costly damage to the current collector and the overhead line. Such replacement of the sliding strip without visible signs of damage is a sanctioned practice in the rail vehicle operation system. There are no specific, clear criteria for making such a decision. Otherwise, the cause of replacement is various types of damage. The most common types of damage to carbon sliding strips are sliding strip cracks, part of the sliding strips peel-off, the top layer of tape torn off, material melting as a result of arc discharge, and tape breakage [13].

The reason for replacing the sliding strip, in this case, exceeds the recommended strip thickness. In the event of minor defects that do not result in the belt losing the current-receiving capacity, e.g., by rubbing the belt edge, there is no need to replace the sliding strips. Such damages are caused by the impact on the hard points of the catenary and it is often assumed that minor surface damages may not exceed 30% of the surface of the carbon strip. However, if there is any major damage, then the strip should be replaced, because it may damage the overhead line.

In the case of replacement following damage, there are steps to be undertaken following certain evaluation criteria such as:

- Material melting as a result of arcing and damages caused by arcing;
- Detachment of a piece of carbon strip;
- Cracks of a sliding strip;
- The top layer of a carbon strip is peeling off.

## 2. Materials and Methods

In the field of materials used for pantograph sliding strips, many studies have been carried out to ensure that its use enables the longest possible reliable operation and is also ecological or has the least impact on the environment. The effect of the material was analyzed by testing contact strips made of pure carbon [14], copper-impregnated carbon [15], iron-base [16], and carbon composite [17] and by verifying cases where lubricants have a positive effect [18]. The studies focused on the effects of single parameters prove how difficult it is to develop a validated real case model that relates wear to material properties and operating conditions [19].

In China, trains are generally divided into three classes: low-speed trains with operation speeds lower than 160 km/h, medium−speed trains with operation speeds of 160–250 km/h, and high−speed trains with operation speeds of 250–350 km/h. In order to obtain high wear resistance and good economic benefits, different types of sliders and contact wires are used in these three speed classes of the railway system. Sintered alloy strips, metal−impregnated carbon shoes, and carbon shoes are used in low-, medium-, and high-speed trains, respectively. The cost of producing a carbon slide is three times that of a sintered alloy strip, whereas the cost of producing a metal-impregnated carbon slide is three times that of a carbon sliding strip.

Each country has a different railway infrastructure, therefore different speeds may be allowed. Additionally, the weather conditions differ and different construction solutions are used, so it is important that material tests are carried out in real conditions.

This section describes the materials for sliding strips used in Poland and describes the test method, which focuses on strip damages caused by material defects.

### 2.1. Materials of a Sliding Strip

#### Copper and Carbon Composites Sliding Strips

Currently, in Poland, a carbon composite is used for the production of sliding strips used in the current collector. Formerly, the sliding strips were made of copper. However, the disadvantage of the copper strips was the relatively high abrasive wear of the overhead catenary and itself. The mutual friction and wear of both elements are increased due to the known phenomenon of bad cooperation of elements of the same material. Therefore, it was decided to replace the used electrolytic sliding strips with carbon composite strips. These materials are carbon-metal composites. Most often they are sintered carbon in the form of graphite with copper and other metals such as tin, antimony, etc. The exact chemical composition and production technology are the manufacturer’s know—how. The percentage of components is selected depending on the type of traction used.

When modeling the pantograph, appropriate parameters of its individual elements should be assumed. The sliding strip and the upper arm can be modeled as flexible bodies and the remaining parts as rigid bodies. The parameters of individual elements are presented, among others, in the work [11]. Exemplary material parameters of pantograph components that can be used for modeling are presented below [11]:

- graphite contact strip (e.g., carbon fiber, Density: 2.7 g/cm^3^, Elastic Modulus:23 GPa, Poisson’s Ratio: 0.1)
- collector head and upper arm (e.g., aluminum alloy, Density: 2.7 g/cm^3^, Elastic Modulus: 72 GPa, Poisson’s Ratio: 0.33, Yield Strength: 280 MPa)
- lower arm and coupling rod base frame (e.g., Stainless steel, Density: 7.85 g/cm^3^, Elastic Modulus: 200 GPa, Poisson’s Ratio: 0.3, Yield Strength: 250 MPa)

Of course, the presented values are only an example, and so, for instance, the density of the graphite sliding strip made of the MY7A2 material is 2.5 g/cm^3^, and for the CY280 material, it is 1.6 g/cm^3^.

Detailed parameters of the selected materials used for the current collector sliding strips according to [20] are presented below:

- Material: CY280 (plain carbon graphite, lead-free)
    Typical Running Current: 6 A/mm
    Typical Static Current: 1.0 A/mm
    Specific Resistance: 38 μohm
    Density: 1600 kg/m^3^
    Transverse Bend Strength: 35 MN/m^2^
    Hardness Scaleroscope: 75
- Material: MY7A2 (metalized material CY280 for higher strength and lower resistance, lead-free)
    Typical Running Current: 14 A/mm
    Typical Static Current: 2.3 A/mm
    Specific Resistance: 5 μohm
    Density: 2500 kg/m^3^
    Transverse Bend Strength: 70 MN/m^2^
    Hardness Scaleroscope: 75
- Material: CY3TA (plain carbon graphite, lead-free)
    Typical Running Current: 6 A/mm,
    Typical Static Current: 1.0 A/mm
    Specific Resistance: 38 μohm
    Density: 1700 kg/m^3^
    Transverse Bend Strength: 30 MN/m^2^
    Hardness Scaleroscope: 85
- Material: MY7A (metalized material CY3TA for higher strength and lower resistance, lead-free)
    Typical Running Current: 10 A/mm
    Typical Static Current: 2.0 A/mm
    Specific Resistance: 10 μohm
    Density: 2400 kg/m^3^
    Transverse Bend Strength: 75 MN/m^2^
    Hardness Scaleroscope: 90

The material MY7A2 is important due to the subject of this paper, which is used, among others, in sliding strips for DSA150, AKP4E, and 5ZL current collectors. In further research carried out in this article, MY7A2 material was used.

Tests of wear and damage to sliding strips for the same carbon composite were carried out for three different types of the current collector. The sliding was made of MY7A2 material, whereas the difference was the shape of the sliding strips, the method of its assembly, as well as the operating characteristics of the current collector–traction network, which was caused by the use of three different types of pantographs in terms of construction. A detailed description of the methodology of the conducted simulation tests is provided in Section 2.2.

### 2.2. Examination of Sliding Strips Damage Caused by Material Defects

The research included three types of current collectors: AKP−4E, 5ZL, and 150 DSA. They had carbon sliding strips made of MY7A2 material. By testing the same material on different types of current collectors, it was possible to check what impact the wear and damage of the sliding strips have, among others, on the way of their assembly, i.e., whether the damage to the sliding strips depends on the type of current collector and what is the share of damage caused by material defects [21]. 

The tests were carried out in the conditions of the normal operation of rail vehicles and covered a period of about one year. Average monthly temperatures ranged from 1.9 °C to 22.9 °C. The tested sliding strips were mounted on three different types of pantographs, of which about 10% were DSA 150 collectors, about 30% 5ZL, and 60% AKP 4E. In total, after the initial data processing, 803 measurements of overlay thickness were obtained, which belonged to the replacement cycle with at least three measurements. The measurement cycle should be understood as the time from the installation of the sliding strip with a new one, where the thickness was 45 mm, until its next replacement. This article pays particular attention to cases of strip replacement due to material defects. The reasons for such replacement include:

- Cracks in the sliding strips;
- Extraction of the sliding strips material;
- Detaching a fragment of the sliding strips.

The crack in the sliding strips material, apart from material defects, may be caused by the current collector hitting a small obstacle on the overhead line, e.g., hitting an improperly installed section isolator, contact wire hanger element, contact wire hanger element, and an environmental element such as branches and ice nuggets. Breakage of the sliding strips may cause damage to the overhead line or detachment of the sliding strips.

Extraction of the material is mainly caused by defects in the material and, as in the case of a crack, it may be caused by a mechanical impact against an element of the external environment.

The reason for the detachment of a fragment of material is, above all, the irregularities that occurred during the design and production of the sliding strips.

Due to the lack of registration of the reasons for the replacement of the sliding strips, in the first step, it was necessary to develop the methods to identify the reason for the replacement of the sliding strips. For this purpose, decision rules were developed to determine whether the collector strip was replaced due to, among others, material defects. The exemplary algorithms for technical conditions and for replacement causes were shown in a different paper by authors [13]. Equation (1) shows a case of inferring sliding strip replacement due to material defects.

(1) $$\begin{array}{cc} N=1\Leftrightarrow \left(\left(L_{i+1}\right.\right. & \left.=L_{i}\right)\wedge \left(T_{i+1}=T_{i}\right)\wedge \left(Wn_{i}\neq 1\right)\\ \; & \left.\wedge \left(\left(Gn1_{i}-Gn1_{i+1}<0\right)\vee \left(Gn2_{i}-Gn2_{i+1}+1<0\right)\right)\right)\\ \; & \wedge (N1+N3=0)\wedge ((Gn1>33)\vee (Gn2>33)) \end{array}$$

where:

*N*—replacement of the sliding strip due to detachment of a fragment of the sliding strip, material extraction or burning of the sliding strip
*Wn*—replacement of the pantograph sliding strip
*L*—the locomotive number
*N*—op the pantograph number
*T*—the type of pantograph
*Gn*1—thickness of the first carbon sliding strip
*Gn*2—thickness of the second carbon sliding strip
*N*1—replacement of the sliding strip due to even wear of the sliders
*N*3—replacement of the sliding strip due to uneven wear of the sliders
*i*—the measure number

Due to the lack of documentation of the causes of the replacement of the current collector components, the above Equation (1) allows for the identification of the reason for the replacement other than the typical materials wear of the sliding strips and the uneven wear of the sliding strips, which would be caused by the uneven pressure of the collector to the overhead line. Thus, apart from the exchange caused by material defects, the developed decision rules also take into account material melting as a result of arcing and damages caused by arcing. Sparks and burns on the sliding strips mainly occur when the outside temperature is below 0 °C. By eliminating the cases of replacing the collector strip that took place in the winter period, it can be assumed that most of the replacements of the sliding strips for which the logical value of 1 was obtained from Equation (1) concern replacements caused by material defects.

The prepared set of data necessary to conduct the analyses used in this publication contains:

1. Number of inspection in the measurement cycle;
2. Type of current collector;
3. Current collector number;
4. Information about position (front/rear pantograph);
5. The difference in the thickness of the sliding strips (for both sliding strips);
6. Inspection date;
7. Reason for the replacement of the sliding strips; only cases showing the replacement caused by material defects (extraction, detachment, cracking) and overburning of the sliding strips were selected. Then the samples of the winter period from this data set were selected. In this period, significant change in thickness of the sliding strips and the replacement were probably caused by burnout, not due to material defects.

The test results were presented in the form of box and whisker plots, Pareto–Lorenz diagrams, statistical summaries, and the F-test with two samples for variance.

## 3. Results

From the conducted research, charts and tables were prepared in order to visualize the relationship between the analyzed data.

In the beginning, an analysis of the variance between the data sets concerning the replacement of the sliding strips caused by its damage (material defects and burnout of the sliding strips caused by an electric arc) was carried out in order to check whether the sets differ statistically from each other. Then, the analysis of variance was performed using the F-test for different types of pantographs. In next step box-and-whisker plots of the thickness of both tested contact strips was prepared, as well as the averaged value was calculated of the thickness for both contact strips during each measurement, distinguishing between the type of current collector.

The relationship between the frequency of damage and the thickness of the sliding strips in the form of histograms together with the Pareto–Lorenz plot, as well as in the form of summaries was prepared.

Also a descriptive statistics, where the results of, among others, parameters such as: mean, standard error, median, standard deviation, sample variance, range, minimum, maximum, confidence level (95.0%) was prepared.

In the beginning, two theories were tested:

1. The theory regarding the possibility of qualifying to the group of “damage to the collector strip” presented in Equation (1), both cases of replacement of the collector strip due to material defects and overburning of the collector strip.
2. The theory of differences in damage characteristics caused by mounting the contact strip on different types of collectors.

### 3.1. Two Sample F–Test for Variance

**Theorem** **1.** *The group of damage to the collector strip includes both—damage caused by material defects and burns to the collector strip. The average thickness of the sliding strips during its damage caused by material defects, i.e., exfoliation of the material (peeling off the top layer of a strip), detach a fragment of the sliding strips or a crack of a strip, in statistical terms it is comparable to the average thickness of the sliding strips during replacement caused by its overburning.*

As part of the research, the correlation between the data sets on the thickness of the sliding strips during its replacement with a new one was checked. For this purpose, the following task was formulated: 

Check at the significance level a = 0.05 the hypothesis that two sets including

A—cases of replacement of the sliding strip due to material defects

B—cases of replacement of the sliding strip due to damage to the strip

It is characterized by similar differences in the thickness of the sliding strips when replacing it with a new one.

In order to verify this hypothesis, 23 cases were selected representing the replacement of the sliding strips caused by material defects, and 37 cases determining the replacement of the sliding strips caused by its damage (burning, cracking, detachment, exfoliation). For both cases, the variance of the sliding strips thickness was determined for a given set of A and B.

The null hypothesis H_0_: *s*_1_^2^ *= s*_2_^2^ was checked against the alternative hypothesis: H_0_: *s*_1_^2^ *≠ s*_2_^2^, and the empirical value of the Fisher–Snedecor statistic was determined. On this basis, the value of F (Equation (2)) was calculated using the formula:

(2) $$F=\frac{s_{1}^{2}}{s_{2}^{2}}$$

**Proof.**  The test results are summarized in Table 2. In this case, the value of the one-sided F-test (F_0,05;22;36_) was 1.845104737 and turned out to be greater than the calculated F = 1.299954375.

Since F < F _0,05;22;36_ there is no reason to reject the H_0_ hypothesis, we assume that both tested sets are statistically equal. □

**Table 2:** Test F: with two samples for variance for sliding strip replacement due to material defects (A) and strip damage (B).

	*Sliding Strip Thickness*	
	*A*	*B*
Mean	34.08695652	33.87838
Variance	9.287549407	7.14452
Observations	23	37
df	22	36
F	1.299954375	
P (F ≤ f) one-sided	0.236796781	
One-sided F-test	1.845104737	

The correlation between the data on the thickness of the sliding strips during the replacement due to A—material defects and C—overburning of the sliding strips was also checked. Because the result of the one−sided F−test was 2.43792 and is greater than the calculated value of F = 2.186719, it can be concluded that both sets are statistically identical. The results are presented in Table 3.

**Theorem** **2.** *The damage characteristics of carbon sliding strips, measured by the value of the thickness of the strip during its replacement with a new one, are different due to the type of pantograph on which the strip is mounted.*

In order to verify this thesis, an analysis of variance was performed for three types of current collector. The results of the variance test for the AKP−4E and 5 ZL pantographs are presented in Table 4, the results for the DSA 150 and 5 ZL pantograph in Table 5, and for the DSA 150 and AKP−4E pantograph in Table 6.

**Proof.**  The analysis of variance for the AKP−4E and 5ZL pantograph shows that there is no significant difference between them in statistical terms. Both sets can therefore be considered equal. The F−test result in this case was 1.521016, whereas the one−sided F−test was 3.466863. However, the situation is different when comparing the variances of other types of pantographs. □

From the variance test for pantograph DSA 150 and 5ZL, it can be seen that the value of the F index was 12.17465, whereas the one-sided F-test, i.e., F _0.05; 9; 7_ = 3.676675. Since F > F_0.05; 9; 7_ is the basis for rejecting the H_0_ hypothesis, we assume that both tested sets are statistically different.

A significant difference between the values of the F and one-sided F-test was also noticed in the case of the DSA 150 and AKP4E collectors. In this case, the value of the F-test was 8.004289, whereas F_0.05; 9; 18_ = 2.456281.

### 3.2. Box and Whisker Plot 

**Figure 2:**
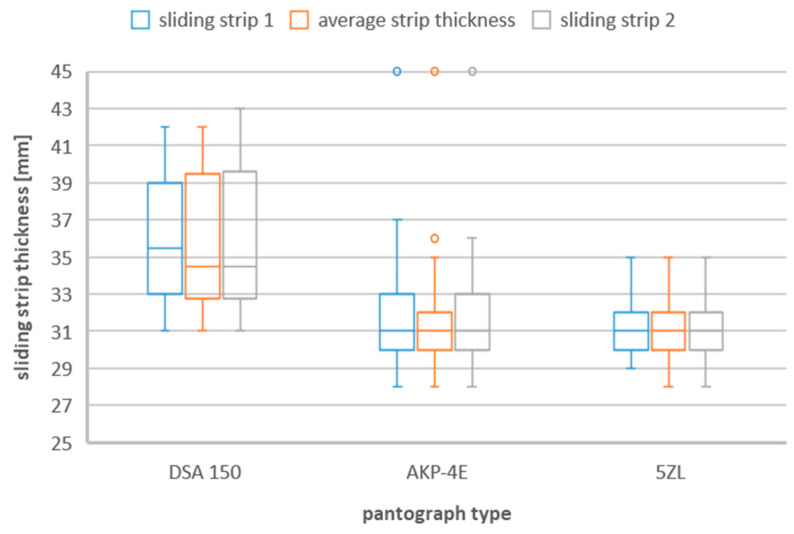
Sliding strip thickness during replacement taking into account the three types of pantographs—DSA 150, 5ZL, AKP−4E.

**Figure 3:**
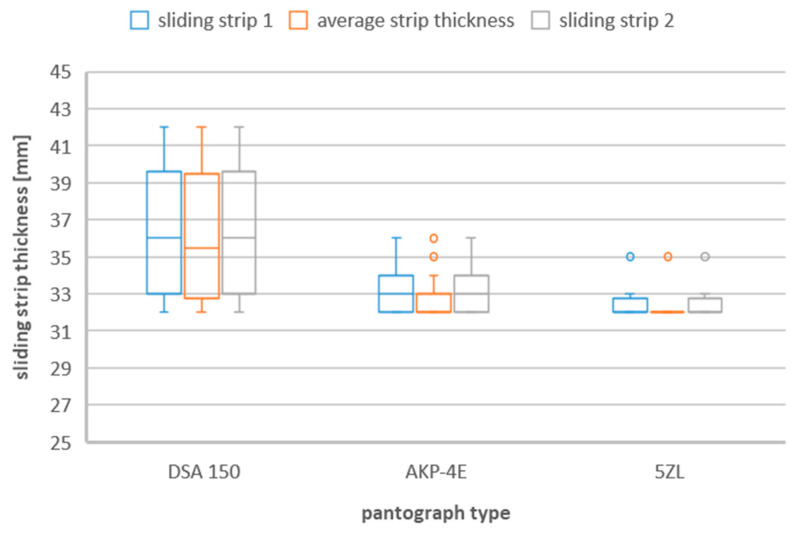
Sliding strip thickness during replacement caused by strip damage taking into account the three types of pantographs—DSA 150, 5ZL, AKP−4E.

**Figure 4:**
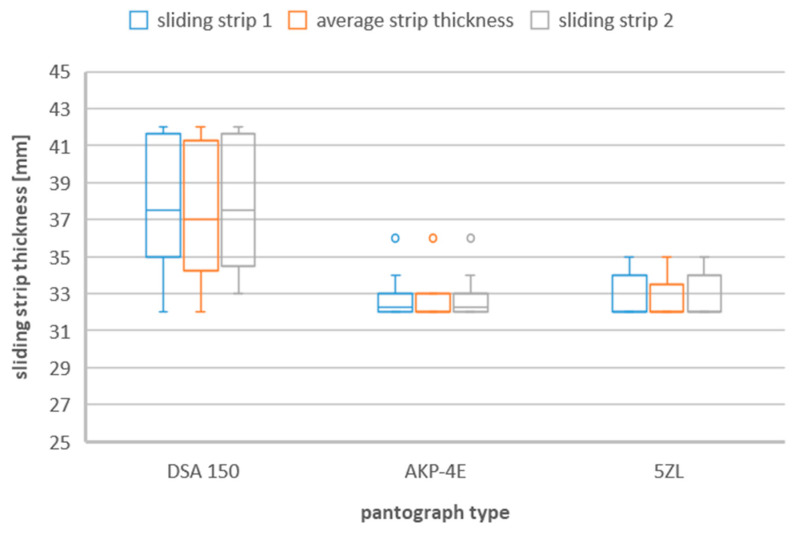
Sliding strip thickness during replacement caused by material defects taking into account the three types of pantographs—DSA 150, 5ZL, AKP−4E.

Since the values of the thickness of the sliding strips during their replacement with a new one differ between different types of current collectors, the list in the form of a box-and-whisker plot is presented in the following figures (Figure 2, Figure 3 and Figure 4).

From Figure 2, due to the type of current collector, we can see differences in the thickness of the collector strip when replacing it with a new one. For the DSA 150 pantograph, this range is much larger than for the other two pantographs. The DSA 150 pantograph is a one-arm (so-called one-half) pantograph and unlike the AKP 4E and 5ZL pantographs (four-arm, so-called two-half pantographs), they are lighter and have lower lateral stiffness. Half pantographs are commonly used on all new electric rail vehicles, so it can be said that these pantographs are used at higher speeds than single half pantographs such as AKP 4E or 5ZL. A higher vehicle speed may therefore be the reason for a greater range of sliding strip thickness during replacement.

This range for DSA 150 pantograph is from 31 mm to 43 mm. The lower quartile is 32.75 mm, whereas the upper quartile for the second slide contact strip is 39.63 mm. In this case, the median is 34.5 mm.

In the case of the 5ZL pantograph, the maximum thickness range was noted for strip 1 and it ranges from 28 mm to 35 mm. The lower quartile is 30 mm, whereas the upper quartile is 32.38 mm for the second sliding strip.

The minimum thickness of the AKP−4E pantograph strip is 28 mm, whereas the maximum is 37 mm. In the case of this type of pantograph, there were also cases of replacement of the contact strip with a new one, when its thickness was 45 mm. These measurements, on the other hand, represent single measurement points above 1.5 times the value of the upper quartile. For this type of collector, for the sliding strip No. 1 and 2 the upper quartiles were, respectively, 37 mm and 36 mm.

### 3.3. Pareto–Lorenz Diagrams

**Figure 5:**
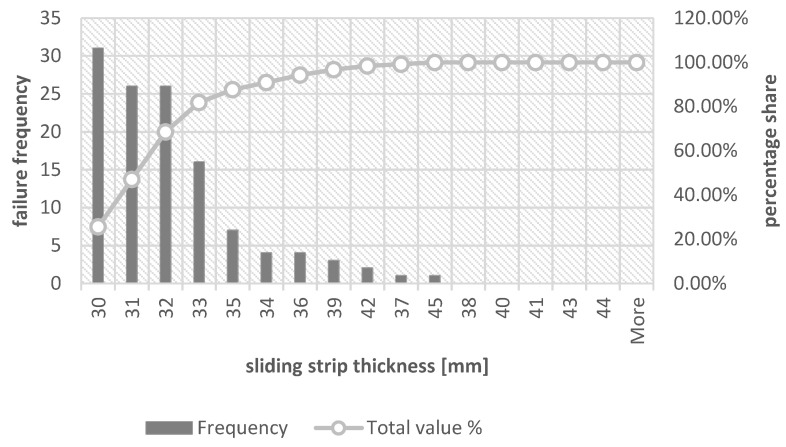
Sliding strip thickness histogram during replacement—cases included all reasons for replacement.

**Figure 6:**
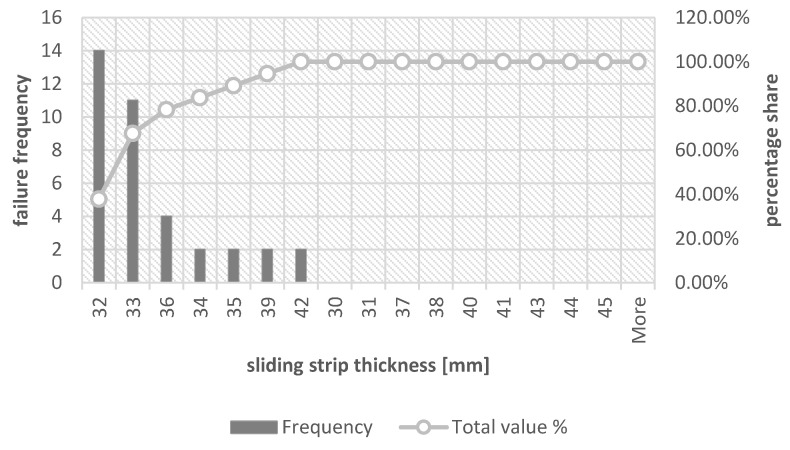
Sliding strip thickness histogram during replacement—cases included replacement caused by strip damage.

**Figure 7:**
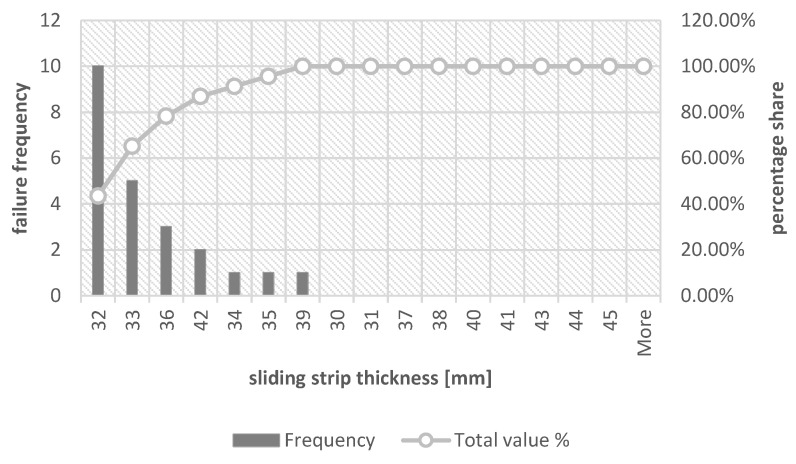
Sliding strip thickness histogram during replacement—cases included replacement caused by material defects (the average temperature in the month of measurement did not exceed 6.5 °C).

In order to visualize the thickness of the sliding strips for which its replacement is most often performed, histograms were drawn together with the Pareto–Lorenz diagram. Figure 5 shows a graph considering all the reasons for replacing the sliding strip, Figure 6 shows the thickness of the collector strip during replacement due to its failure, whereas Figure 7 is a more detailed version of the earlier drawing (Figure 6) because it shows a histogram with a Pareto–Lorenz plot for the group of collector strips during its replacement due to material defects.

As can be concluded from the presented graphs, the most common thickness of the sliding strips during replacement is 30 mm, accounting for 25.62% of all cases, then 31 mm and 32 mm. In this range of thickness, as much as 68.6% of all sliding strips are replaced.

In the case of replacement due to material defects, it can be observed that the most common replacement takes place when the thickness of the collector strip is 32 mm. It constitutes 37.84% of all cases, whereas together with a thickness of 33 mm it is a total of 67.57%. For the same thicknesses, 65.22% of all exchanges caused by material defects can be observed, respectively, for the thickness of 31 mm it is 43.48% and for the thickness of 33 mm it is 21.74%. A tabular summary showing the percentage share of the relationship between the thicknesses of the contact strip during the replacement is presented in Table 7.

According to Theory 1 and its analysis, also in the case of the strip thickness values presented in Table 7, there are no significant differences between the replacement caused by damage to the collector strips and the replacement caused by material defects. The average difference in the replacement rate of the collector strip due to these causes for all collector strip thicknesses is 1.4 percentage points, whereas the maximum difference can be observed for thicknesses different from 33 mm, which is less than 8 percentage points.

### 3.4. Descriptive Statistics

Table 8 shows the test results in the form of descriptive statistics, taking into account the replacement of the sliding strips caused by material defects, distinguishing between three types of pantographs. In the case of replacement of the collector strip due to its damage, in particular material defects, the average thickness of the collector strip in the case of the 5ZL pantograph is 32.6 mm (median 32 mm). This is about 0.5 mm less than the average thickness of the AKP−4E pantograph cap. For the DSA 150 pantograph, it is 36.45 mm (where the median is 36 mm), which is 3.85 mm higher than for the 5ZL pantograph.

The sliding strips mounted on the AKP−4E pantographs are used on average 166.68 days to their damage caused mainly by material defects. The median in this case is 161 days. In the case of 5ZL pantographs, the sliding strips are used for a slightly shorter period. Here, the mean is 148.5 days and the median is 155.5 days. The shortest use of sliding strips is on DSA 150 pantographs, where the average time to failure is 114.8 days, and the median is 100. The minimum number of days of using the sliding strips until it is damaged can be observed in the case of the AKP−4E pantograph, and it is 59 days, whereas the maximum number of days was as many as 270.

## 4. Discussion

The examination of damage to pantograph sliding strips, which was caused by material defects, can be included in a more general group of reasons for the replacement of the strip, i.e., the reasons for replacement caused by sliding strip damage. This group includes, among others, such damage as material exfoliation (peeling off the top layer of a strip), detachment of a fragment of the sliding strips, overburning of the contact strips caused by an electric arc, and cracks of the sliding strips. The thickness range caused by damage during the replacement of the compact strip is 32 to 42 mm.

Undoubtedly, the type of the current collector has an influence on the thickness of the collector strip during the replacement caused by its damage. When analyzing the influence of the pantograph type on the damage characteristic of the sliding strip, it should be noted that the average time to damage the contact strips is the longest in the case of the AKP−4E pantograph, and the shortest in the case of the DSA 150 pantograph. In the case of the AKP−4E pantograph, it ranges from 59 to 270 days, so this range is as high as 211 days. The smallest time range in which the sliding strip is damaged can be observed in the case of the DSA 150 pantograph. This range is 142 days.

Summing up, it should be noted that the type of pantograph on which it is used has an undoubted influence on the damage characteristics of the carbon sliding strip, whereas the damage caused by material defects can be included in a more general group−the group of strip damage, which also includes carbon strip burn marks−overburning caused by arcing.

In order to more accurately assess whether the type of damage to the contact strip is related to the given thickness of the contact strip during replacement and the number of used days, it would be necessary to record the reason for replacing the contact strip with a new one each time. By changing the currently existing system, it would be possible to investigate the relationship between such damage types as detachment of strip fragment, peeling off the top layer of a strip, cracks of the strip and overburning of the contact strip, the thickness of the collector strip, the frequency of damage with the pantograph type, and the time of use until damage.

The results of conducted analyses may be used to build a preventive maintenance strategy for the pantographs. The models of wear propagation can be extended by the parameters of the cost and repair time becoming the basis for estimating the costs of operation and maintenance.

## Figures and Tables

**Table 1:** Parameters of the current collector type DSA150, AKP4E, 5ZL.

	Unit	DSA150	AKP4E	5ZL
**Maximum operating speed**	km/h	160	125	160
**Type of construction**		single−arm	diamond−shaped	diamond−shaped
**Material**		Steel/Aluminum	Steel/Aluminum	Steel/Aluminum
**Weight (without support insulators)**	kg	125	380	380
**Highest lifting height**	mm	3000	2300	2300
**Height in the lowest position, without insulator**	mm	550	365	365
**Sliding length**	mm	1280	1270	1100
**Glider length**	mm	1450/1600/1950	2030	1950
**Rated voltag:**	kV	15/25 (other power supplies are also implemented)	3	3
**Rated intensity**	A	800/1000 (other power supplies are also implemented)	800–1200	1200–1800
**Static pressing force**	90	70–120 (adjustable)	90	90
**Drive type**		Pneumatic	Spring	Spring
**Drive power**	bar	5	>4	>4
**Lifting time**	s	<10 (adjustable)	6–12	6–12
**Lowering time**	s	<10 (adjustable)	5–10	5–10

**Table 3:** Test F: with two samples for variance for sliding strip replacement due to material defects (A) and strip overburning (C).

	*Sliding Strip Thickness*	
	*A*	*C*
Mean	34.08696	33.64286
Variance	9.287549	4.247253
Observations	23	14
df	22	13
F	2.186719	
P (F ≤ f) one−sided	0.07356	
One-sided F−test	2.43792	

**Table 4:** Test F: with two samples for variance—AKP−4E and 5ZL pantograph.

	Pantograph Type	
	AKP-4E	5ZL
Mean strip thickness	33.10526	32.5
Variance	1.738304	1.142857
Observations	19	8
df	18	7
F	1.521016	
P (F ≤ f) one−sided	0.29567	
One-sided F−test	3.466863	

**Table 5:** Test F: with two samples for variance—pantograph DSA 150 and 5ZL.

	Pantograph Type	
	DSA 150	5ZL
Mean strip thickness	36.45	32.5
Variance	13.91389	1.142857
Observations	10	8
df	9	7
F	12.17465	
P (F ≤ f) one−sided	0.001673	
One-sided F−test	3.676675	

**Table 6:** Test F: with two samples for variance—pantograph DSA 150 and AKP−4E.

	Pantograph Type	
	DSA 150	AKP-4E
Mean strip thickness	36.45	33.10526
Variance	13.91389	1.738304
Observations	10	19
df	9	18
F	8.004289	
P (F ≤ f) one−sided	0.000103	
One-sided F−test	2.456281	

**Table 7:** Thickness of the sliding strip at the time of replacement to a new one depending on the type of damage.

Sliding Strip Thickness	Replacement Frequency [%]		
	All Reasons for Replacement	All Strip Damages	Damages Caused by Material Defects
30	25.62	0.00	0.00
31	21.49	0.00	0.00
32	21.49	37.84	43.48
33	13.22	29.73	21.74
34	3.31	5.41	4.35
35	5.79	5.41	4.35
36	3.31	10.81	13.04
37	0.83	0.00	0.00
38	0.00	0.00	0.00
39	2.48	5.41	4.35
40	0.00	0.00	0.00
41	0.00	0.00	0.00
42	1.65	5.41	8.70
43	0.00	0.00	0.00
44	0.00	0.00	0.00
45	0.83	0.00	0.00

**Table 8:** Descriptive statistics for the AKP-4E, 5ZL, DSA 150 pantograph for cases of sliding strip replacement due to material defects.

	Pantograph Type	Days After Replacement	Inspection Number	Quarter	Strip Thickness 1	Strip Thickness 2
Mean	AKP−4E	166.68	5.04	3.16	33.04	33.14
	5ZL	148.50	4.90	3.10	32.60	32.60
	DSA 150	114.80	4.80	1.80	36.45	36.45
Standard error	AKP−4E	11.79	0.36	0.21	0.25	0.26
	5ZL	18.54	0.59	0.38	0.34	0.31
	DSA 150	13.17	0.59	0.25	1.18	1.14
Median	AKP−4E	161	5	4	33	33
	5ZL	155.5	5	3.5	32	32
	DSA 150	100	4	2	36	36
Standard deviation	AKP−4E	58.94	1.79	1.07	1.23	1.32
	5ZL	58.62	1.85	1.20	1.07	0.97
	DSA 150	41.65	1.87	0.79	3.73	3.61
Sample variance	AKP−4E	3473.56	3.21	1.14	1.52	1.74
	5ZL	3436.72	3.43	1.43	1.16	0.93
	DSA 150	1734.40	3.51	0.62	13.91	13.03
Range	AKP−4E	211	5	3	4	4
	5ZL	165	5	3	3	3
	DSA 150	142	6	2	10	10
Minimum	AKP−4E	59	3	1	32	32
	5ZL	83	3	1	32	32
	DSA 150	73	3	1	32	32
Maximum	AKP−4E	270	8	4	36	36
	5ZL	248	8	4	35	35
	DSA 150	215	9	3	42	42
Confidence level (95.0%)	AKP−4E	24.33	0.74	0.44	0.51	0.54
	5ZL	41.94	1.33	0.86	0.77	0.69
	DSA 150	29.79	1.34	0.56	2.67	2.58

## Data Availability

The data presented in this study are available on request from the corresponding author.
